# Author Correction: Structural phase transition in NH_4_F under extreme pressure conditions

**DOI:** 10.1038/s42004-025-01457-7

**Published:** 2025-02-24

**Authors:** Umbertoluca Ranieri, Christophe Bellin, Lewis J. Conway, Richard Gaal, John S. Loveday, Andreas Hermann, Abhay Shukla, Livia E. Bove

**Affiliations:** 1 Dipartimento di Fisica, Università di Roma La Sapienza, Rome, Italy; 2 Centre for Science at Extreme Conditions and School of Physics and Astronomy, University of Edinburgh, Edinburgh, UK; 3 Institut de Minéralogie, de Physique des Matériaux et de Cosmochimie, Sorbonne Université, CNRS UMR 7590, Paris, France; 4 Department of Materials Science and Metallurgy, University of Cambridge, Cambridge, UK; 5 Advanced Institute for Materials Research, Tohoku University, Sendai, Japan; 6 Laboratory of Quantum Magnetism, Institute of Physics, École Polytechnique Fédérale de Lausanne, Lausanne, Switzerland

**Keywords:** Chemical physics, Structure of solids and liquids, Raman spectroscopy

Correction to: *Communications Chemistry* 10.1038/s42004-024-01309-w, published online 30 September 2024

In the Acknowledgements section the following sentence was added: We acknowledge the European Synchrotron Radiation Facility for provision of synchrotron radiation facilities, and we would like to thank Michael Hanfland and Mohamed Mezouar for assistance in using the beamlines ID15b and ID27. The original article has been corrected.

