# Supplementary material for: Structural phase transition in NH₄F under extreme pressure conditions
Source: Commun Chem. 2024 Sep 30;7:220. doi: 10.1038/s42004-024-01309-w (PMC11443071; doi:10.1038/s42004-024-01309-w)
Supplement: Supplementary file 2 — Supplementary Information file [file 42004_2024_1309_MOESM2_ESM.pdf]

Supplementary Materials for

Structural Phase Transition in NH_4F under Extreme Pressure Conditions

Umbertoluca Ranieri[1,2], Christophe Bellin[3], Lewis J. Conway[4,5], Richard Gaal[6],

John S. Loveday[2], Andreas Hermann[2], Abhay Shukla[3], Livia E. Bove[1,3,6]

[1]Dipartimento di Fisica, Università di Roma La Sapienza, Piazzale Aldo Moro 5, 00185 Rome, Italy

[2]Centre for Science at Extreme Conditions and School of Physics and Astronomy, University of Edinburgh, Peter Guthrie Tait Road, EH9 3FD Edinburgh, United Kingdom

[3]Institut de Minéralogie, de Physique des Matériaux et de Cosmochimie, Sorbonne Université, CNRS UMR 7590, 5 Place Jussieu, 75005 Paris, France

[4]Department of Materials Science and Metallurgy, University of Cambridge, 27 Charles Babbage Road, Cambridge CB30FS, United Kingdom

[5]Advanced Institute for Materials Research, Tohoku University, Sendai, 980-8577, Japan

[6]Laboratory of Quantum Magnetism, Institute of Physics, École Polytechnique Fédérale de Lausanne, CH-1015 Lausanne, Switzerland

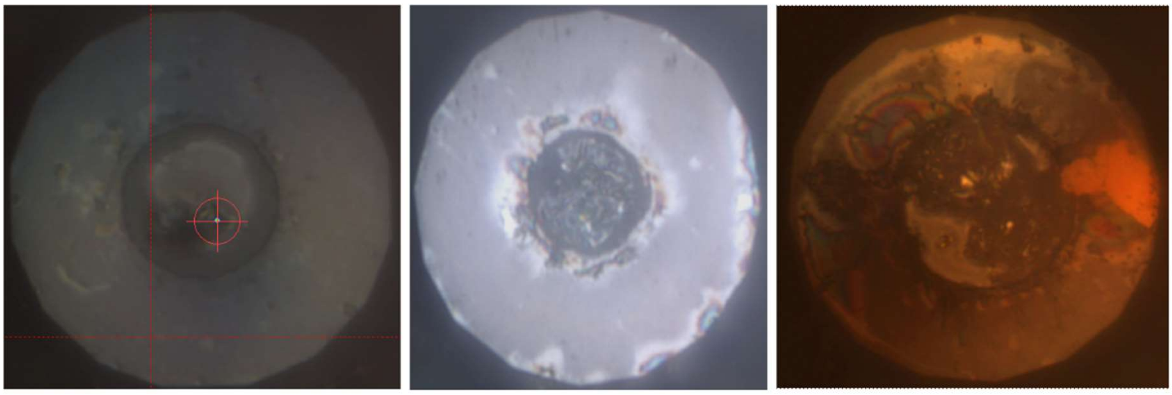

Supplementary Figure 1: Photomicrographs of the three samples of this work: DAC#1 at 6 GPa (left), DAC#2 at 7 GPa (middle), and DAC#3 at 4 GPa (right). The full scale of each of the three images is about 100 µm.

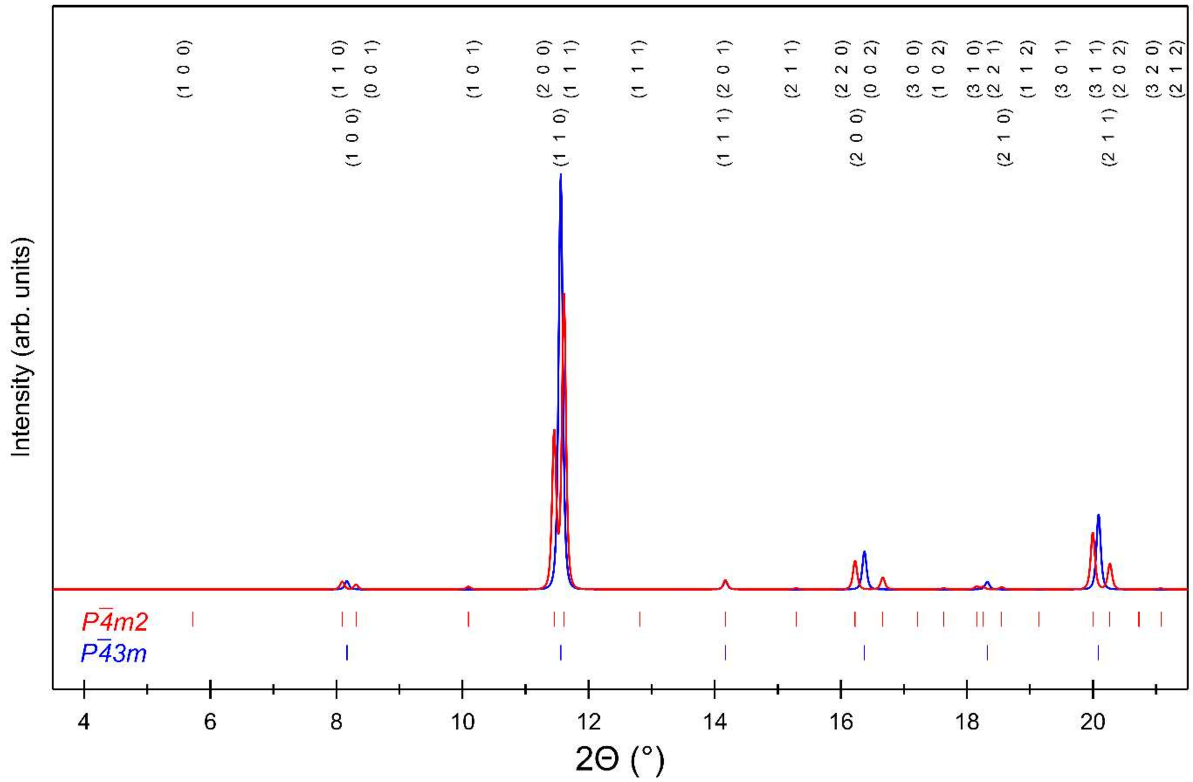

Supplementary Figure 2: Calculated X-ray diffraction patterns for perfect powders of NH_4F-*P-4m2* (a=4.11 Å and c=2.83 Å) and NH_4F-*P-43m* (a=2.88 Å), both at 50 GPa (λ= 0.4101 Å). Tick marks indicate the positions of the Bragg reflections and Miller indices are also reported.

Supplementary Table 1: DFT-optimised crystal structures of the three NH_4F phases encountered in this work. The pressure-dependent F-displacement magnitudes are highlighted in bold; zero displacement would correspond to z_F=0 for NH_4F-IIIt and to z_F=½ for NH_4F-VIII. The corresponding crystallographic information files are provided with this manuscript.

**Phase**	**Crystal system**	**Space Group**	**P (GPa)**	**Lattice parameters**	**Volume (Å³)**	**Atom Site x y z**
NH_4F-III	cubic	*P-43m* (n. 215)	20	a=b=c=3.064 Å<br>α=β=γ=90°	28.77	N 1b ½ ½ ½<br>H 4e 0.3043 0.3043 0.3043<br>F 1a 0 0 0
NH_4F-IIIt	tetragonal	*P-4m2* (n. 115)	80	a=b=4.010 Å<br>c=2.722 Å<br>α=β=γ=90°	43.76	N 1c ½ ½ ½<br>N 1d 0 0 ½<br>H 4k 0.2917 ½ 0.7128<br>H 4j 0 0.2083 0.2872<br>F 2g 0 ½ **0.0367**
NH_4F-VIII	tetragonal	*P4/nmm* (n. 129)	120	a=b=3.899 Å<br>c=2.552 Å<br>α=β=γ=90°	38.80	N 2a 0 0 0<br>H 8i 0 0.2106 0.7706<br>F 2c 0 ½ **0.4226**

Supplementary Table 2: DFT-optimised crystal structure of the hypothetical NH_4F-IIIt phase with space group *P-42m* discussed in ref. 1.

**Phase**	**Crystal system**	**Space Group**	**P (GPa)**	**Lattice parameters**	**Volume (Å³)**	**Atom Site x y z**
NH4F-IIIt	tetragonal	*P-42m* (n. 111)	80	a=b=2.835 Å<br>c=2.722 Å<br>α=β=γ=90°	21.88	N 1b ½ ½ ½<br>H 4n 0.708 0.708 0.287<br>F 1a 0 0 0

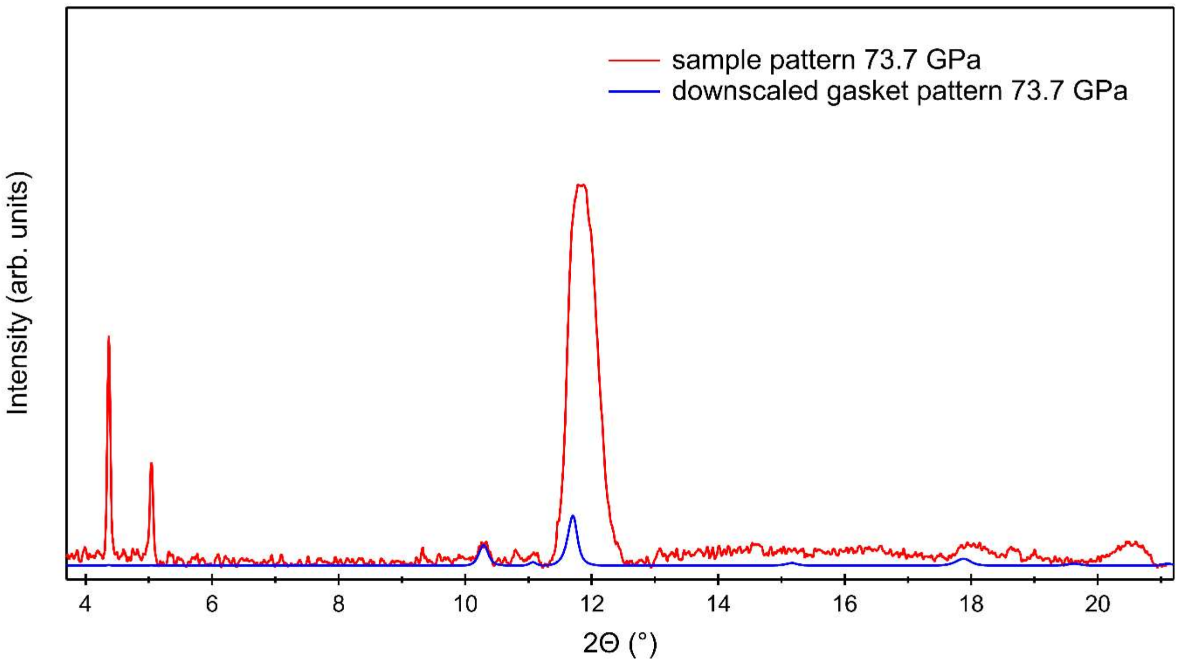

Supplementary Figure 3: Example of X-ray diffraction pattern collected on the Re gasket, together with the sample pattern at the same pressure of 73.7 GPa (λ= 0.4101 Å). The intensity of the Re pattern was arbitrarily scaled so that the Re (1 0 0) reflection at 10.3° has about the same magnitude in the two patterns. One can see that the Re (1 0 1) reflection at 11.7° overlaps with the NH_4F signal but it is much weaker.

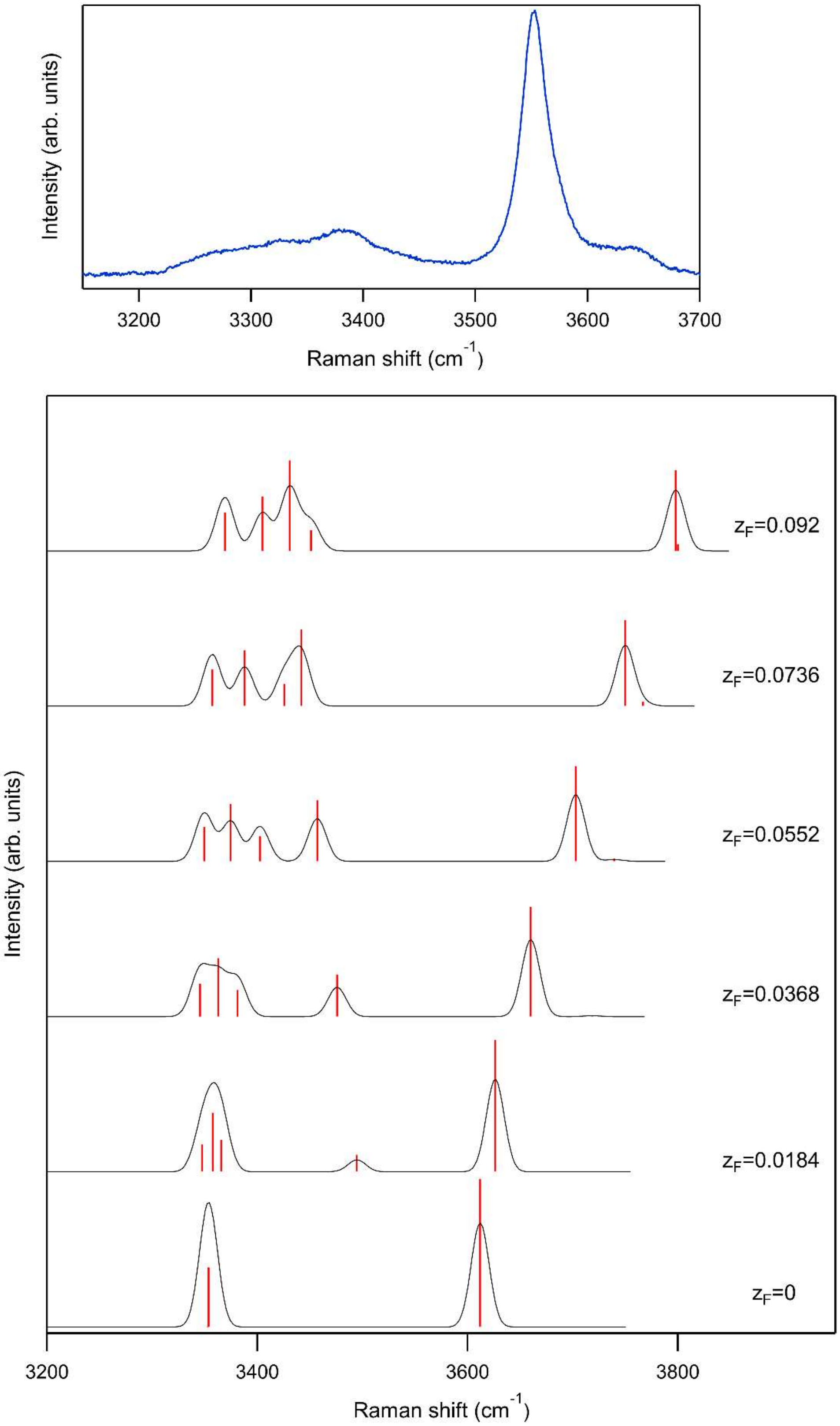

Supplementary Figure 4: Experimental Raman spectrum at 114 GPa (top) and calculated Raman spectra of NH_4F-*P-4m2* at 114 GPa for different z-displacements of the F atom in the unit cell (bottom). All spectra were Gaussian-broadened with the same width. Spectra were calculated with steps of

0.00184 in the coordinate z_F. Thirty spectra were simulated in total. The best agreement with the experimental spectrum was estimated to be for z_F=0.04048. This is based on i) the difference in frequency between features and ii) the shapes obtained by merging individual mode intensities upon applying the Gaussian broadening. Note that there is a high-frequency peak which is calculated to be very weak but which is clearly seen experimentally (100 cm^{-1} right of the main peak).

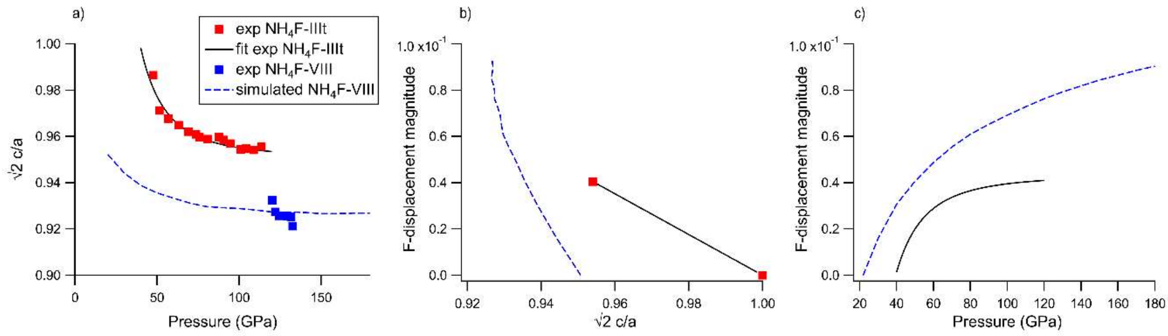

Supplementary Figure 5: (a) √2 c/a ratio as a function of pressure, (b) F-displacement magnitude as a function of √2 c/a ratio and (c) F-displacement magnitude as a function of pressure. Legend of panel a also applies to panels b and c. Symbols represent experimental data, dashed lines represent DFT calculations. The black curve of panel a is a fit to the experimental data for NH_4F-IIIt. The black curve of panel b is a linear interpolation between the values z_F=0 at 40 GPa and z_F=0.04048 at 114 GPa. The black curves of panels a and b were used to deduce the black curve of panel c. Finally, the black curve of panel c was used to impose z_F in the simulations of the Raman spectra of phase IIIt at various pressures (reported in Fig. 4 of the main text).

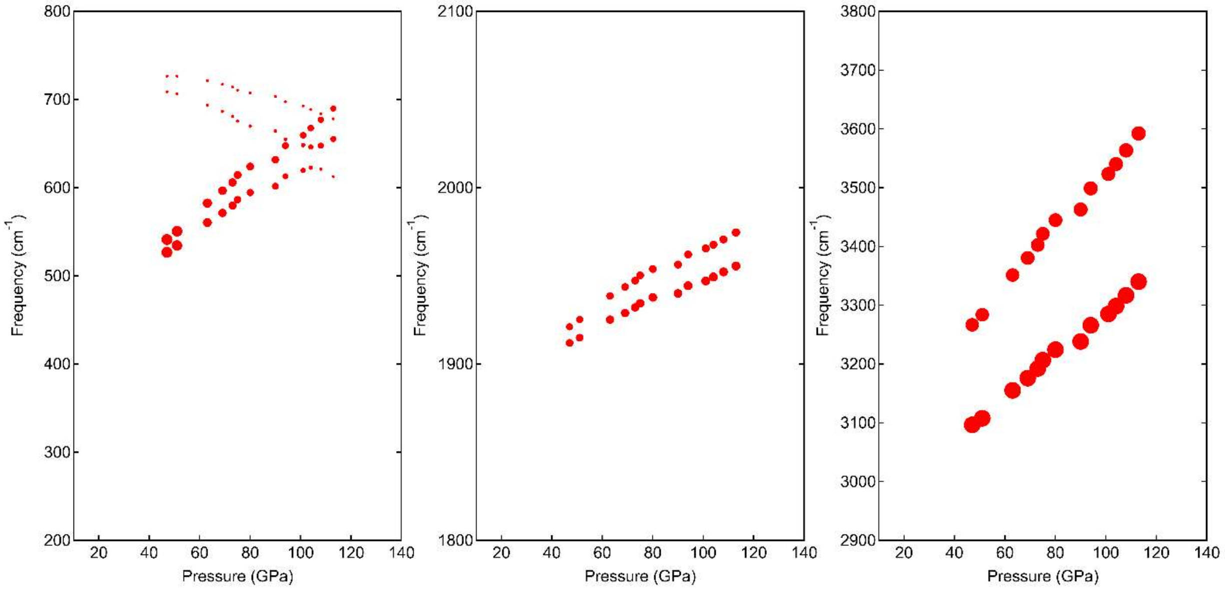

Supplementary Figure 6: Theoretical Raman lattice mode (left), bending region (center), and stretching region (right) frequencies for NH_4F-*P-42m*. The size of the dots is given by the intensity of the Raman peaks.

Supplementary Table 3: Mode assignment for Raman-active Γ phonons of the *P-43m* phase, point group Td, as calculated from lattice dynamics and assessed from displacement eigenvector. Calculated frequencies and intensities from DFT lattice dynamics are given for 100 GPa. Acoustic modes have zero frequency and are omitted.

**Symmetry**	**ν (cm^{-1})**	**Relative intensity**
T2	663.9	0.0029
T2	1370.3	0.0003
E	1959.0	0.0316
T2	3303.5	1
A1	3547.1	0.8148

Supplementary Table 4: Mode assignment for Raman-active Γ phonons of the *P-4m2* phase, point group D2d, as calculated from lattice dynamics and assessed from displacement eigenvector. Calculated frequencies from DFT lattice dynamics are given for 100 GPa. Acoustic modes have zero frequency and are omitted.

**Symmetry**	**ν (cm^{-1})**	**Relative intensity**
E	150.9	3E-07
A	472.6	4.84E-05
B	513.1	6E-08
E	542.7	0.0005
B	633.3	0.0015
E	659.2	0.0029
E	763.9	0.0016
E	810.8	3.2E-07
B	1349.8	3.24E-06
E	1365.7	0.0006
E	1548.8	1.99E-06
B	1575.9	5.12E-05
A	1637.6	2.36E-05
A	1816.3	0.0008

A	1949.5	0.0276
A	1961.5	0.0244
E	3299.1	0.5620
B	3305.7	0.5595
E	3318.7	0.4782
A	3420.0	0.3376
A	3581.1	1
B	3647.9	0.0083

Supplementary Table 5: Mode assignment for Raman-active Γ phonons of the *P4/nmm* phase, point group D4h, as calculated from lattice dynamics and assessed from displacement eigenvector. Calculated frequencies from DFT lattice dynamics are given for 100 GPa. Acoustic modes have zero frequency and are omitted.

**Symmetry**	**ν (cm^{-1})**	**Relative intensity**
Eg	191.1	0.0008
B2g	392.5	0.0005
A1g	456.4	0.0080
Eg	735.4	0.0003
Eg	761.5	1.24E-05
B2g	1370.9	0.0001
Eg	1525.2	2.12E-05
A1g	1981.2	0.0207
B1g	2034.3	0.0199
B2g	3371.0	0.4184
Eg	3418.1	0.6769
A1g	3594.0	1

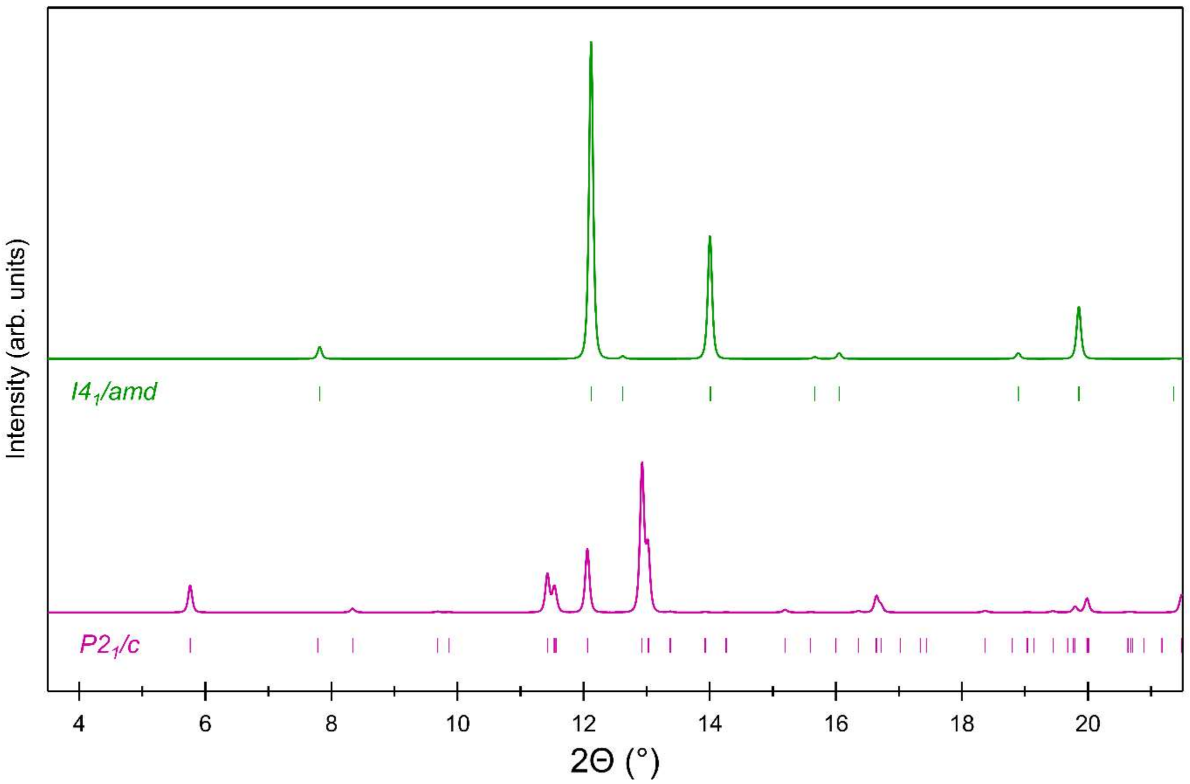

Supplementary Figure 7: Calculated X-ray diffraction patterns for perfect powders of NH_4F-*I4₁/amd* and NH_4F-*P2₁/c* at 130 GPa (λ= 0.4101 Å). Tick marks indicate the positions of the Bragg reflections. Detailed structural information on these two structures is given in ref. 2.

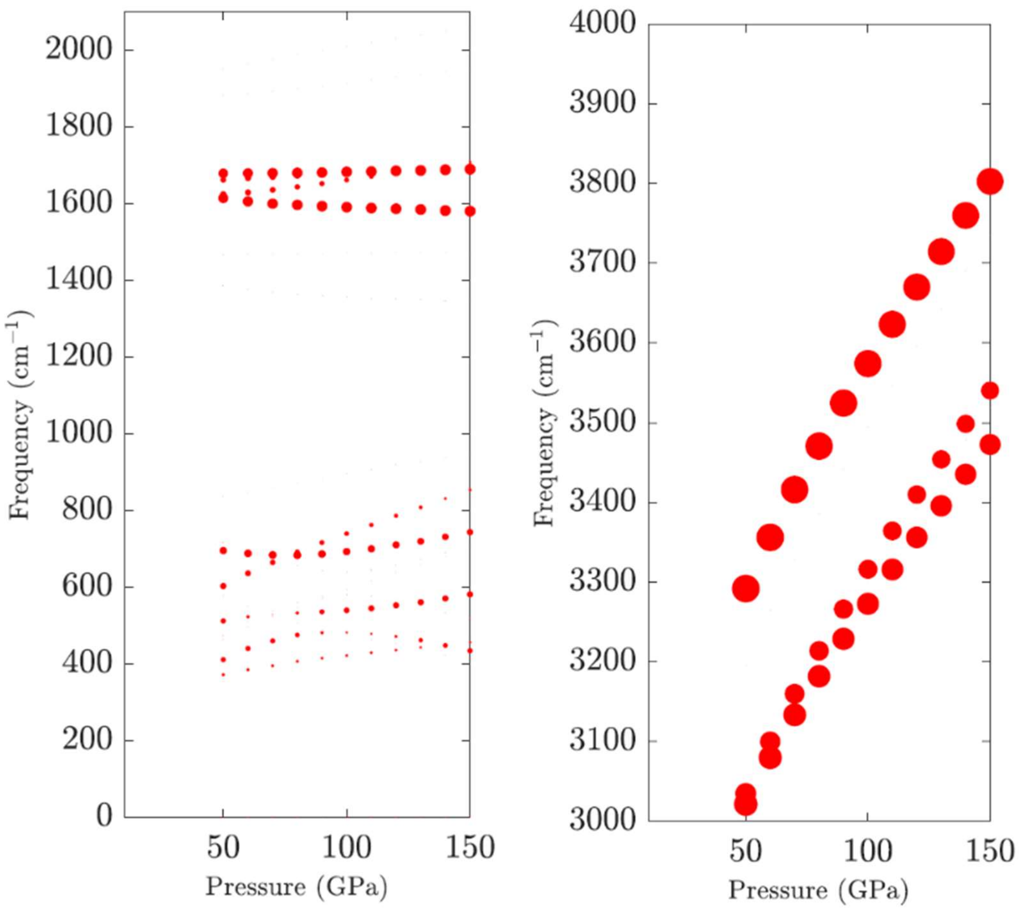

Supplementary Figure 8: Theoretical Raman lattice mode (left) and stretching region (right) frequencies for NH_4F-*I4₁/amd*. The size of the dots is given by the intensity of the Raman peaks.

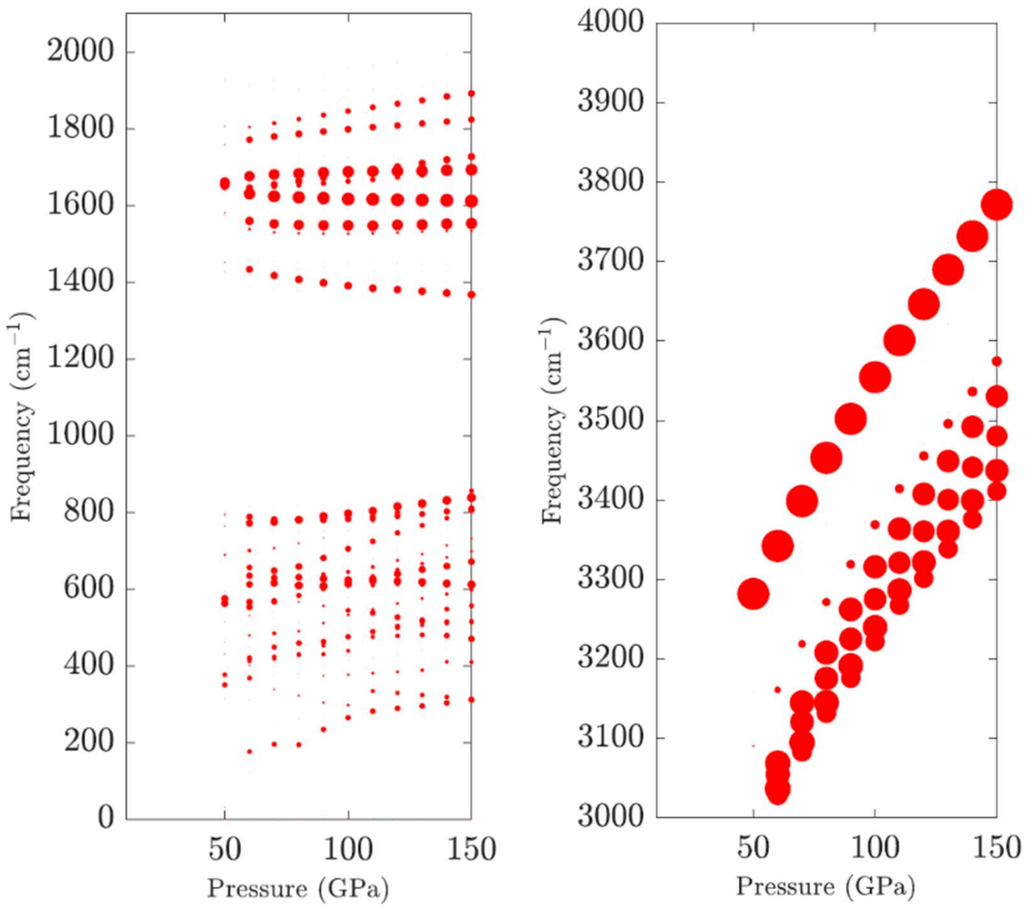

Supplementary Figure 9: Theoretical Raman lattice mode (left) and stretching region (right) frequencies for NH_4F-*P2₁/c*. The size of the dots is given by the intensity of the Raman peaks.

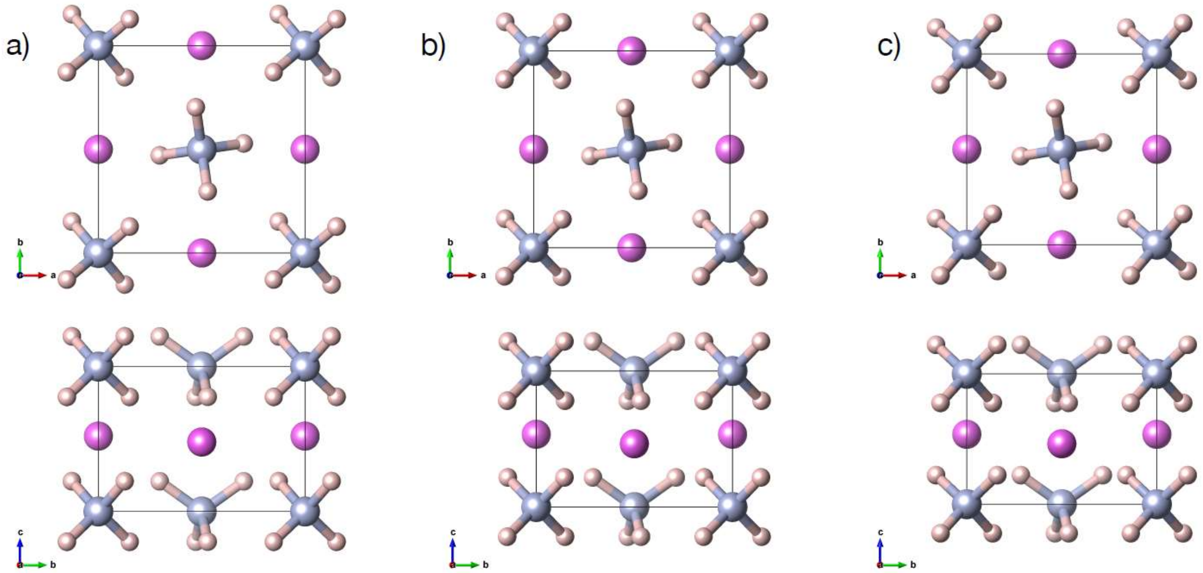

Supplementary Figure 10: Crystal structures of the saddle points along the NEB transition paths from phase III to phase VIII. a–c) Structures at 50/100/150 GPa, respectively. Top/bottom row are top/side views, all to the same scale.

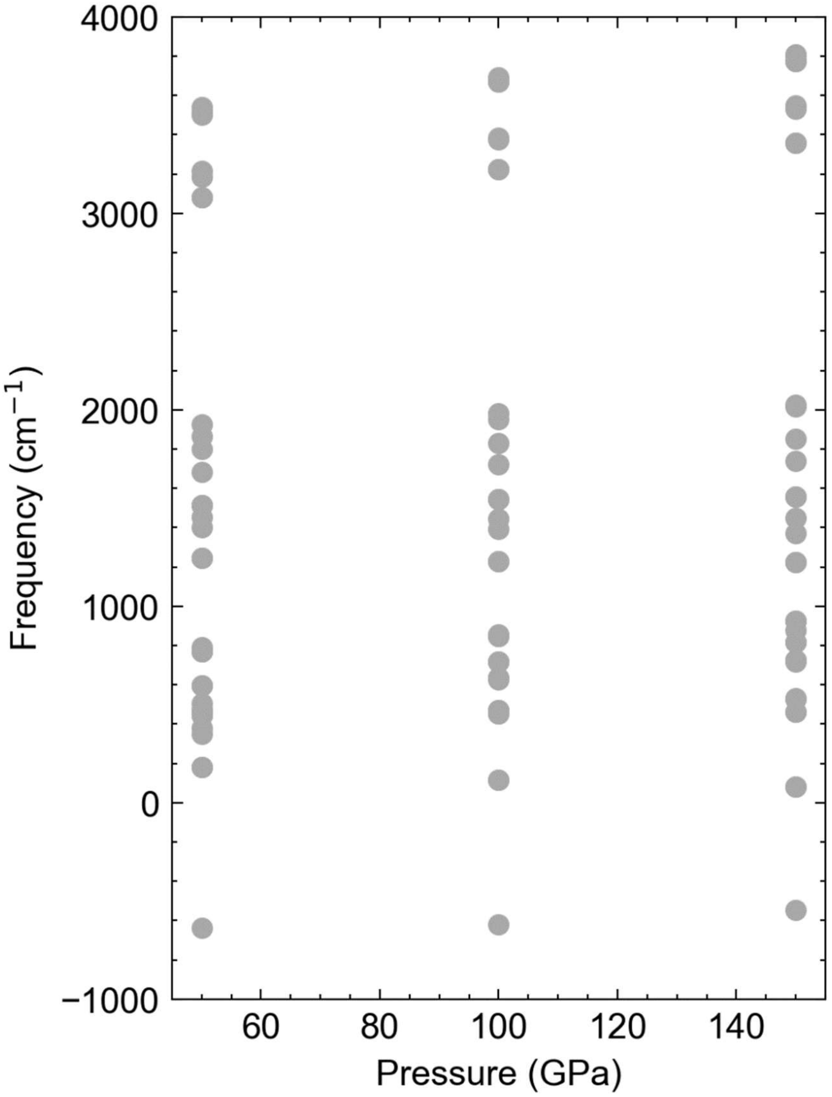

Supplementary Figure 11: Theoretical Raman frequencies at the saddle points along the NEB transition paths from phase III to phase VIII, at 50/100/150 GPa.

Supplementary Table 6: Crystal structure information of the saddle points along the NEB transition paths from phase III to phase VIII. All structures are in the tetragonal space group *P-4* (n. 81). The corresponding crystallographic information files are provided with this manuscript.

**Pressure (GPa)**	**Lattice parameters**	**Volume (Å^3)**	**Atom Site x y z**
50	a=b=4.100 Å<br>c=2.864 Å	48.13 Å^3	N 1a 0 0 0<br>N 1c ½ ½ 0<br>H 4h 0.1520 0.1277 0.2090<br>H 4h 0.5287 0.2972 0.2081<br>F 2g 0 ½ 0.4796
100	a=b=3.893 Å<br>c=2.689 Å	40.74 Å^3	N 1a 0 0 0<br>N 1c ½ ½ 0<br>H 4h 0.1477 0.1447 0.2180<br>H 4h 0.5338 0.2897 0.2177<br>F 2g 0 ½ 0.4650
150	a=b=3.771 Å<br>c=2.578 Å	36.66 Å^3	N 1a 0 0 0<br>N 1c ½ ½ 0<br>H 4h 0.1377 0.1613 0.2229<br>H 4h 0.5358 0.2852 0.2227<br>F 2g 0 ½ 0.4638

Supplementary References

[1] C. Bellin *et al.* Phys. Rev. B 96, 094110 (2017).

[2] L. J. Conway, K. Brown, J. S. Loveday, A. Hermann, J. Chem. Phys. 154, 204501 (2021).
