# Supplementary material for: Structural phase transition in NH₄F under extreme pressure conditions
Source: Commun Chem. 2024 Sep 30;7:220. doi: 10.1038/s42004-024-01309-w (PMC11443071; doi:10.1038/s42004-024-01309-w)
Supplement: Supplementary file 3 — Description of Additional Supplementary Files [file 42004_2024_1309_MOESM3_ESM.pdf]

Description of Additional Supplementary Files

File name: Supplementary Data 1

Description: DFT-optimised crystal structure of NH_4F-III at 20 GPa

File name: Supplementary Data 2

Description: DFT-optimised crystal structure of NH_4F-IIIt at 80 GPa

File name: Supplementary Data 3

Description: DFT-optimised crystal structure of NH_4F-VIII at 120 GPa

File name: Supplementary Data 4

Description: Crystal structure information of the saddle point along the NEB transition path from phase III to phase VIII at 50 GPa

File name: Supplementary Data 5

Description: Crystal structure information of the saddle point along the NEB transition path from phase III to phase VIII at 100 GPa

File name: Supplementary Data 6

Description: Crystal structure information of the saddle point along the NEB transition path from phase III to phase VIII at 150 GPa
